# Research on Characteristics of Broadband Acoustic Sensor Based on Silicon-Based Grooved Microring Resonator

**DOI:** 10.3390/mi12111338

**Published:** 2021-10-30

**Authors:** Jiangong Cui, Yaxin Yu, Xiaoxia Chu, Rongyu Zhao, Min Zhu, Wendong Zhang, Guojun Zhang

**Affiliations:** State Key Laboratory of Dynamic Testing Technology, North University of China, Taiyuan 030051, China; jgcui@nuc.edu.cn (J.C.); s1906103@st.nuc.edu.cn (Y.Y.); b200607@st.nuc.edu.cn (X.C.); s2006241@st.nuc.edu.cn (R.Z.); s2006170@st.nuc.edu.cn (M.Z.); wdzhang@nuc.edu.cn (W.Z.)

**Keywords:** grooved waveguide, photoacoustic coupling, broadband, acoustic sensor

## Abstract

In order to meet the requirements of having a small structure, a wide frequency band, and high sensitivity for acoustic signal measurement, an acoustic sensor based on a silicon-based grooved microring resonator is proposed. In this paper, the effective refractive index method and the finite element method are used to analyze the optical characteristics of a grooved microring resonator, and the size of the sensor is optimized. The theoretical analysis results show that, when the bending radius reaches 10 μm, the theoretical quality factor is about 10^6^, the sensitivity is 3.14 mV/Pa, and the 3 dB bandwidth is 430 MHz, which is three orders of magnitude larger based on the sensitivity of the silicon-based cascaded resonator acoustic sensor. The sensor exhibits high sensitivity and can be used in hydrophones. The small size of the sensor also shows its potential application in the field of array integration.

## 1. Introduction

Acoustic measurement is an important research means in non-destructive testing, underwater anti-submarine monitoring, health observation and biomedical imaging [1,2,3,4]. At present, traditional optical acoustic sensors based on fiber Fabry–Perot interferometer (FPI) [5,6], polymer fiber [7], or fiber Bragg grating (FBG) [8] have the advantages of high sensitivity and anti-electromagnetic interference compared with piezoelectric hydrophones, playing an important role in acoustic monitoring. However, most of these optical hydrophones rely on the amount of mechanical deformation to realize acoustic signal sensing and cannot avoid the limitations of self-resonance and narrow frequency.

In contrast, the acoustic signal testing technology based on optical microcavity, by virtue of excellent photoacoustic coupling characteristics, can avoid the limitation of self-resonance and narrow frequency and can expand the frequency range by 2–3 orders of magnitude and the sound wave detection frequency to MHz so that the scope of application can be greatly expanded and so that the device can be integrated into a chip, reaching the sub-centimeter-squared level, while ensuring its detection sensitivity. Sensors based on optical microcavities are often used in biochemical sensing [9], gas detection [10], refractive index sensing [11], and acoustic wave detection [12]. For acoustic wave detection, in order to achieve broadband and high-sensitivity acoustic signal detection, acoustic sensors combining highly sensitive and integrated microring resonators and low Young’s modulus polymers have been proposed and improved. Reference [13] described an acoustic sensor based on a polymer microresonator with a quality factor of up to 10^5^ and a frequency band response of up to 40 MHz. Reference [14] introduced an acoustic sensor based on a microring resonator, which realized 12 MHz sound wave detection, and its sensitivity reached 35 mV/KPa. Reference [15] analyzed a silicon-based acoustic wave sensor based on acoustic membrane resonance and realized acoustic wave sensing at 1–150 MHz. These studies show that the acoustic signal testing technology based on optical microcavity has great application potential in broadband acoustic monitoring.

Therefore, this paper proposes a broadband acoustic sensor using silicon-based grooved microring resonators. Through theoretical analysis and simulation analysis, the photoacoustic coupling mechanism and acoustic sensing characteristics of the resonator are discussed, and the results show that it has good performance in acoustic sensing.

## 2. Theory and Principles

### 2.1. Principle of Microring Resonator

**Figure 1:**
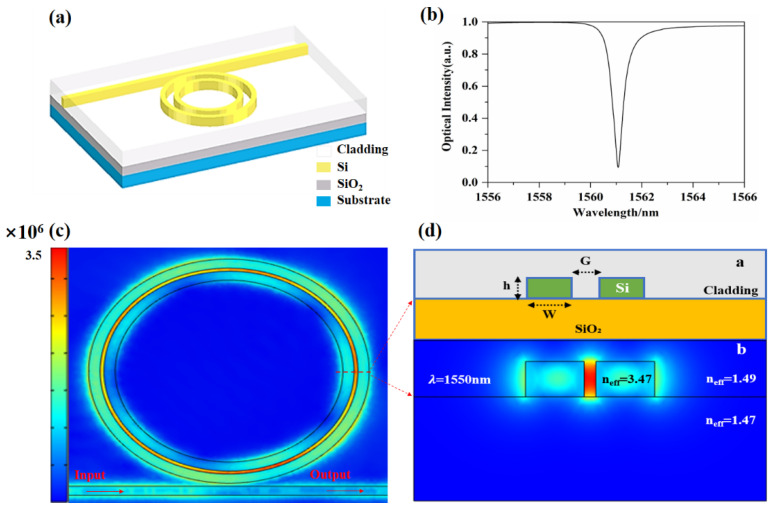
(**a**) Schematic diagram of the structure, (**b**) transmission line of the microring resonator (coupling gap is 100 nm), (**c**) resonant state mode distribution diagram of the resonator cavity, and (**d**) cross-sectional structure diagram and mode distribution diagram.

A diagram of the acoustic sensor structure based on the grooved microring resonator proposed in this paper is shown in Figure 1a. The grooved waveguide is composed of a straight strip waveguide and two ring silicon waveguides. The groove between the silicon waveguides and its upper cladding layer are filled with a polymer, and the silicon dioxide buffer layer is below the silicon waveguide. Figure 1c is a light field distribution diagram that satisfies the resonance state. The straight strip waveguide serves as the input and output ports of the resonator at the same time. When light is input from the input port of the straight waveguide, it is coupled with the curved waveguide of the groove microring resonator via the evanescent wave, and the lightwave that meets the resonance conditions causes resonance in the ring and propagates back and forth in the ring. The light that does not resonate couples with the straight waveguide through the gap. At this time, the normalized transfer function of the microring resonator, *T*, is expressed as follows [16]:

(1) $$T=\frac{{\left|\tau \right|}^{2}-2\alpha \left|\tau \right|cos\theta +\alpha^{2}}{1-2\alpha \left|\tau \right|cos\theta +\alpha {\left|\tau \right|}^{2}}$$

wherein τ represents the transmission coefficient of the coupling zone, α represents the transmission loss factor of the microring, $\theta =\frac{2\pi n_{eff}}{\lambda }L$ represents the phase accumulated by the light propagating in the microring for a circle, λ represents the resonance wavelength, L represents the circumference of the microring, and $n_{eff}$ represents the effective refractive index of the waveguide.

When the optical path of the light beam traveling around the boundary of the geometric structure meets an integer multiple of the wavelength, interference enhancement occurs. The ring resonance equation can be expressed as follows:

(2) $$2\pi Rn_{eff}=m\lambda$$

wherein *R* represents the radius of the microring and *m* represents the number of resonance stages.

The extinction ratio of the output spectrum of the microring resonator, *ER*, is expressed as follows:

(3) $$ER=20\log_{10}\left(\frac{1-\alpha \left|\tau \right|}{1+\alpha \left|\tau \right|}\right)\cdot \left(\frac{\left|\tau \right|+\alpha }{\left|\tau \right|-\alpha }\right)$$

wherein it can be obtained that the extinction ratio of the microring resonator reaches positive infinity when |τ|=α. This condition is critical coupling. At this time, a perfect extinction ratio can be achieved, which is beneficial to the measurement of the spectral drift of the sensor. 

The quality factor of the microring is an important parameter to measure the microring resonator, expressed as follows:

(4) $$Q=\frac{2\pi Rn_{g}\sqrt[{\pi }]{\alpha \tau }}{\lambda \left(1-\alpha \tau \right)}$$

wherein $n_{g}=n_{eff}-\lambda \frac{dneff}{d\lambda }$ represents the group refractive index. From the expression, the quality factor is proportional to the radius of the ring.

### 2.2. Photoacoustic Coupling Effect

When the microring resonator is used in an acoustic sensor, the applied sound wave generates a stress field, and the stress can be calculated by the following equation:

(5) $$\varepsilon =\frac{-\left(1+v\right)\left(1-2v\right)P}{E}$$

wherein v represents the Poisson’s ratio of the material, E represents the Young’s modulus of the material, and P represents the sound pressure generated by the acoustic signal.

Under the influence of sound waves, the sound pressure of the sound waves changes the refractive index of the material of the microring resonator, thereby affecting the effective refractive index of the system. The relationship between the refractive index of the material in different directions and the magnitude of the stress can represent the elasto-optical effect [17]:

(6) $$\{\begin{array}{c} n_{x}=n_{0}-p_{11}\sigma_{x}-p_{12}\left(\sigma_{y}-\sigma_{z}\right)\\ n_{y}=n_{0}-p_{11}\sigma_{y}-p_{12}\left(\sigma_{x}-\sigma_{z}\right)\\ n_{z}=n_{0}-p_{11}\sigma_{z}-p_{12}\left(\sigma_{x}-\sigma_{y}\right) \end{array}$$

wherein $n_{x,y,z}$ represents the refractive index of the material in different directions, $n_{0}$ is the refractive index of the material when there is no sound pressure, $p_{11}$ and $p_{12}$ are the elastic-optical coefficient of the material, and $\sigma_{x,y,z}$ represents the magnitude of the sound pressure in different directions.

According to the optical waveguide theory, the equivalent refractive index method is adopted to obtain the effective refractive index of the grooved waveguide affected by the elasto-optical effect. The relationship between the refractive index change in the microring and the strain of the resonator is as follows [18]:

(7) $$\frac{△n}{n_{0}}=-\frac{n_{0}^{2}\varepsilon }{2}\left[p_{12}-v\left(p_{11}+p_{12}\right)\right]\;$$

When the external sound pressure changes, it causes the medium to interact with the evanescent wave. The effective refractive index of the waveguide changes due to the stress field imposed by the sound pressure change, which changes the resonance mode of the microring resonator and causes drifting of the resonance wavelength of the resonator. The Equation (2) demonstrates that, when the effective refractive index of the mode becomes larger, the resonance wavelength is red-shifted. By measuring the drift of the resonance wavelength, the voltage change can be demodulated to calculate the external sound pressure value.

For acoustic wave sensing, the higher the Q value of the resonator, the higher the sensitivity. The structure, width, and groove gap of the microring resonator all have an impact on the Q value. For acoustic sensors, in order to meet the needs of array integration, the smaller the size of the sensor, the better, so it is necessary to optimize the design of the geometric size of the microring resonator.

## 3. Design and Optimization of Structure

**Figure 2:**
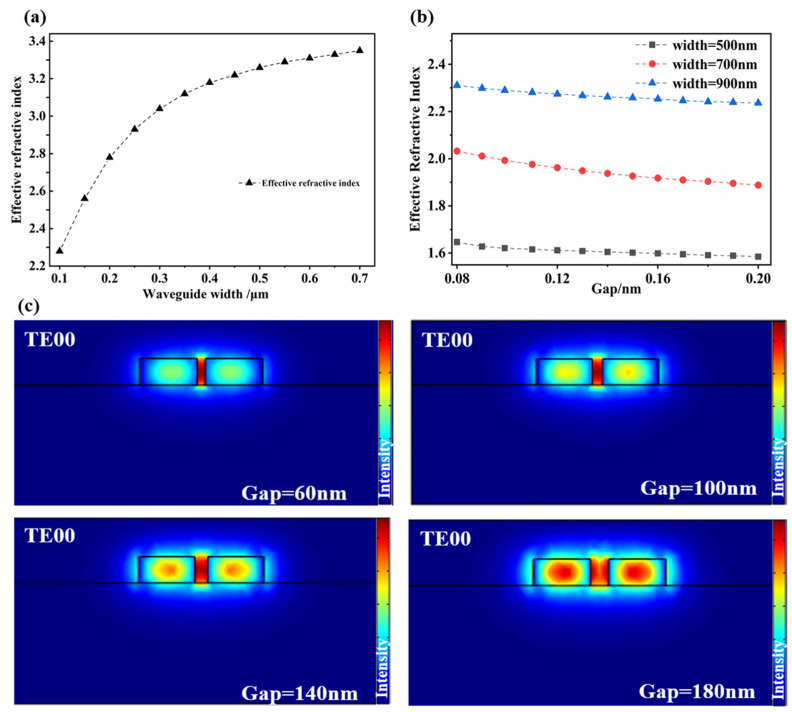
(**a**) The relationship between different waveguide widths and the effective refractive index, (**b**) the relationship between different waveguide widths/gaps and the effective refractive index, and (**c**) the electric field pattern distribution diagram with a width of 450 nm and different gaps.

The curves of waveguides with different widths and the effective refractive index are shown in Figure 2a. Figure 2a shows that, as the width of the waveguide increases, the effective refractive index gradually increases. The influence of the width of the grooved waveguide and the gap between the grooves on the effective refractive index of the grooved waveguide is shown in Figure 2b. Figure 2b shows that, when the width of the grooved waveguide is constant, the width of the groove becomes larger and the effective refractive index becomes smaller; when the width of the groove is constant, the larger the waveguide width and the greater the effective refractive index. In order to obtain a smaller bending radius, the effective refractive index should be as large as possible, so that the bending loss will be relatively small [19]. Therefore, the waveguide should be as wide as possible, and the groove gap should be as narrow as possible. However, the groove gap should not be too narrow, and PMMA should be guaranteed to be spin-coated. In a word, the waveguide width should be 450 nm and the gap should be 100 nm. The waveguide width is 450 nm, and the optical mode distribution corresponding to different gaps is shown in Figure 2c.

In addition, the bending radius of the microring resonator has great influence on the effective refractive index and transmission loss of the waveguide. According to the relationship between bending loss and ring radius in Reference [20], the larger the bending radius, the greater the effective refractive index of the trench waveguide and the smaller the bending loss. Considering the bending loss, in order to obtain a larger Q value, the bending radius should be as large as possible. Therefore, the radius of the microring is selected to be 5 μm.

**Figure 3:**
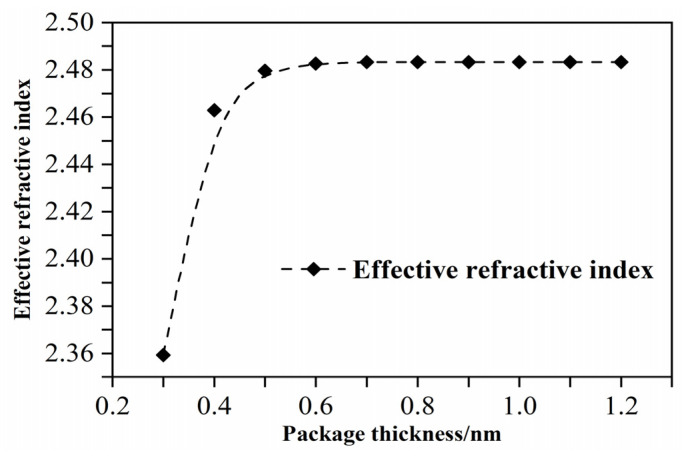
Relationship between different package thicknesses and the effective refractive index.

In order to obtain a better interaction between acoustic waves and light fields, acoustic wave sensors based on optical microcavities, generally, use polymers with a lower Young’s modulus (GPa) as the encapsulation layer. In this study, PMMA was selected as the encapsulation material, and its Young’s modulus was 3 GPa. PMMA has a larger elastic modulus and a smaller Young’s modulus, which is more effective as a coating material. The thickness of the coating layer has an impact on the performance of the sensor, and the coating thickness of PMMA needs to be studied. Figure 3 is the relationship curve between different encapsulation thicknesses and the effective refractive index. After the analysis, the PMMA encapsulation thickness is selected as 0.45 μm. Since the thickness of Si is 220 nm, less than the thickness of PMMA, PMMA can also play a protective role.

Therefore, according to the process and simulation results, the size parameters of the microring resonator are shown in Table 1.

## 4. Analysis of Simulation Results

**Figure 4:**
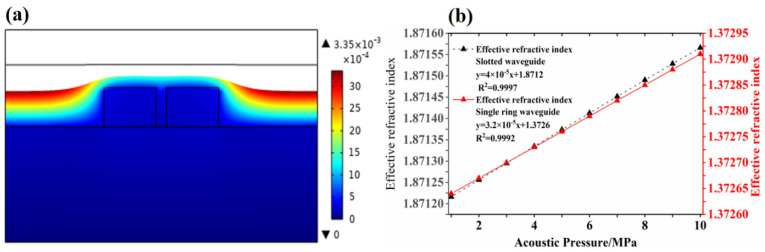
(**a**) Schematic diagram of deformation, and (**b**) the relationship between different sound pressures and the effective refractive index.

Figure 4a shows a deformation diagram of the sensor under the action of 1MPa sound pressure after the simulation analysis. Figure 4b is the relationship between the effective refractive index and the sound pressure calculated after comprehensively considering the effects of deformation and the elasto-optical effects on the effective refractive index of single-ring and grooved waveguides. After linear fitting, the relationship between the effective refractive index and the sound pressure of the grooved waveguide under different sound pressures is expressed as follows:

(8) $$\frac{dn_{eff}}{dP}=4\times 10^{-11}/\mathrm{Pa}$$

wherein $n_{eff}$ represents the effective refractive index and *P* is the sound pressure. The analysis shows that, due to the structural characteristics of the grooved waveguide, the optical field is localized in a small groove, and the refractive index change in the PMMA material caused by the elasto-optical effect is more obvious compared with the effective refractive index of the resonator deformation. Therefore, the relationship between sound pressure and effective refractive index is a positive correlation after linear fitting. In addition, the fitting relationship between the sound pressure and the effective refractive index of the grooved waveguide is larger than that of the single-ring waveguide, and the effective refractive index has a larger value and a larger slope.

**Figure 5:**
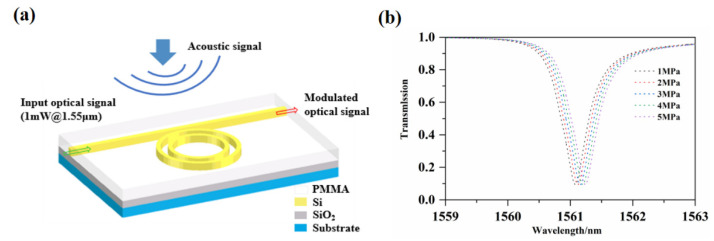
(**a**) Schematic diagram of photoacoustic coupling model and (**b**) spectrum response curve under different sound pressure.

After the simulation analysis, the photoacoustic coupling model of the acoustic sensor is shown in Figure 5a. The input light wavelength is 1.55 μm, and the input light power is 1 mW. In this calculation of the acoustic wave pressure of the output optical power under different sound pressures, the color signal represents the applied acoustic signal, the green arrow represents the input light, and the red arrow represents the output light intensity. A waveguide resonant cavity with a bending radius of 5 μm is selected, with a resonant wavelength of 1561.08 nm as the reference, starting from the sound pressure of 1 MPa; the pressure adjustment step is 1 MPa. The spectrum response curve of the microcavity under different sound pressures is shown in Figure 5b. From the simulation analysis results, it can be concluded that, as the pressure increases, the resonance peak has a 40 pm/MPa linear red shift. According to the resonance Equation (2), the relationship between the resonance wavelength shift and the sound pressure is expressed as follows:

(9) $$\frac{d\lambda_{c}}{dP}=\frac{\lambda_{c}}{n_{eff}}\frac{dn_{eff}}{dP}=0.04\;\mathrm{nm}/\mathrm{MPa}$$

Through a numerical analysis, sensing is achieved by detecting the drift of the resonant wavelength of the resonant spectrum. The requirements for the detection equipment are too high, and the existing equipment may not be able to meet the actual test requirements. In addition to the method of detecting the wavelength shift, the light intensity method can be used as a detection method for detecting the change in light intensity at a specific wavelength for sensing detection of this sensor. The light intensity method relies on the slope of the resonance spectrum curve near the detected wavelength. The greater the slope, the higher the sensitivity. According to the linear relationship between the output light intensity and the wavelength, the size of the sound wave pressure can be demodulated.

## 5. Performance Analysis

### 5.1. Frequency Response

**Figure 6:**
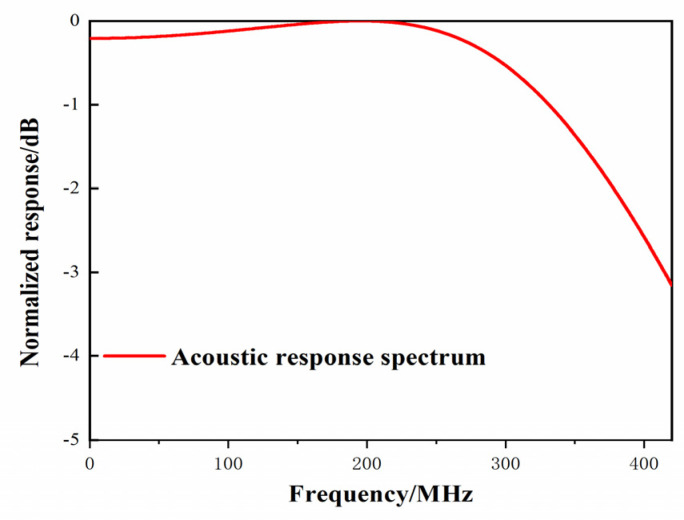
The frequency response of an acoustic sensor based on a slotted waveguide.

In the acoustic signal detection based on the microring resonator, the frequency response of the acoustic sensor is mainly affected by the optical resonance in the microring resonator and the propagation of sound waves. Reference [21] shows that the main factor affecting the frequency response is the Fabry–Perot cavity effect of the sound wave, which be expressed as follows:

(10) $$\left|P_{I}\left(k\right)\right|=\frac{2T}{kl}\sqrt{\frac{\left(R_{0}^{2}+1\right)+4R_{0}\cos k\left(1+2l_{g}\right)\sin^{2}\left(kl/2\right)}{1-2R_{0}R_{1}\cos2k\left(1+l_{g}\right)+{\left(R_{0}R_{1}\right)}^{2}}}$$

wherein $P_{I}\left(k\right)$ represents the normalized frequency response, *k* represents the acoustic wave vector, l represents the thickness of the polymer material, T represents the pressure amplitude transmission coefficient, and $R_{0}$ and $R_{1}$ represent the amplitude reflection coefficient. Given that the thickness of PMMA polymer material is 2 μm, the acoustic impedance of PMMA is 3.2 × 10^6^ Kg/(sm^2^), the acoustic impedance of silicon dioxide is 1.31 × 10^7^ Kg/(sm^2^), and the acoustic impedance of seawater is 1.62 × 10^6^ Kg/(sm^2^). The frequency response can be calculated as shown in Figure 6. The 3 dB bandwidth of the sensor is 430 MHz.

### 5.2. Sensitivity

The performance of acoustic sensors is mainly represented by sensitivity, which is defined as the ratio of transmission intensity to sound wave pressure. The sensitivity of the acoustic sensor is given by the following:

(11) $$S=\frac{dT}{dP}=\frac{dT}{d\lambda }\frac{d\lambda }{dn_{eff}}\frac{dn_{eff}}{dP}\propto Qn_{eff}\frac{dn_{eff}}{dP}$$

wherein *S* is the sensitivity and *T* is the transmission intensity. For an acoustic wave sensor based on a groove microring resonator with a high *Q* value, the transmitted light intensity is related to the position of the resonance wavelength. Therefore, the resonant wavelength shift caused by the sound wave amplifies the change in the transmitted light intensity due to the steep resonant peak curve, thereby increasing its sensitivity.

The sensitivity of the sensor is mainly determined by the *Q* value and the resonance wavelength. According to Equation (4), the bending radius is directly proportional to the quality factor. According to theoretical calculation, when the bending radius reaches 10 μm, the theoretical quality factor is about 10^6^. According to Equation (11), the sensitivity is 3.14 mV/Pa, which is three orders of magnitude larger based on the sensitivity of the silicon-based cascaded resonator acoustic sensor [10], indicating that the sensor proposed in this paper has the advantage of high sensitivity. The performance comparison between the sensor in this paper and the sensor previously studied is shown in Table 2. The comparison shows that the acoustic sensor designed in this research has the advantages of high sensitivity and a wide frequency band.

## 6. Conclusions

This paper proposes an acoustic sensor based on a silicon-based grooved microring resonator. After theoretical analysis and research, it is proved that, when the bending radius reaches 10 μm, the theoretical quality factor is 10^6^, the sensitivity is 3.14 mV/Pa, and the 3 dB bandwidth is 430 MHz. The design and research of sensors with high sensitivity and broadband response have certain reference values and lay the foundation for realizing the application of acoustic sensing in the optical band of communication. In addition, the compatibility with photonic integrated circuit technology causes the sensor to have certain application potentials in array integration.

## Figures and Tables

**Table 1:** Size parameters of microring resonator.

Symbol	Meaning	Value
h	Slot height	220 nm
g	Coupling spacing	100 nm
W	Waveguide width	450 nm
G	Slot width of microring	100 nm
C	Cladding height	450 nm
R	Radius of microring	5 μm
H_SiO2_	SiO_2_ layer height	2 μm

**Table 2:** Sensitivity analysis of microring acoustic wave sensor.

Structure	S (mV/kPa)	Q-Factor	R (μm)	Feq (MHz)
Polymer ring [13]	36.3	1 × 10^7^	30	75
SU8 ring [14]	66.7	8.34 × 10^8^	12	540
Double microring [15]	2.5 × 10^3^	1.24 × 10^6^	5	150
This work	3.14 × 10^6^	3.12 × 10^6^	10	430

## Data Availability

The available data have been stated in the article.
